# How to classify, diagnose, treat and follow-up extragonadal germ cell tumors? A systematic review of available evidence

**DOI:** 10.1007/s00345-022-04009-z

**Published:** 2022-05-12

**Authors:** Christian Winter, Friedemann Zengerling, Jonas Busch, Julia Heinzelbecker, David Pfister, Christian Ruf, Julia Lackner, Peter Albers, Sabine Kliesch, Stefanie Schmidt, Carsten Bokemeyer

**Affiliations:** 1 Urological Practice “Urologie Neandertal”, Erkrath, Germany; 2 Department of Urology and Pediatric Urology, University Hospital of Ulm, Ulm, Germany; 3 UroEvidence@Deutsche Gesellschaft Für Urologie, Berlin, Germany; 4 Department of Urology, Charité Universitaetsmedizin Berlin, Berlin, Germany; 5 Department of Urology and Paediatric Urology, Saarland University Medical Centre, Faculty of Medicine, Saarland University, Homburg, Saar, Germany; 6 Department of Urology, University Hospital Cologne, Cologne, Germany; 7 Department of Urology, Bundeswehrkrankenhaus, Ulm, Germany; 8 Department of Urology, University Hospital Düsseldorf, Medical Faculty, Heinrich-Heine University, Düsseldorf, Germany; 9 Centre of Reproductive Medicine and Andrology, Department of Clinical and Surgical Andrology, University Hospital, Münster, Münster, Germany; 10 Department of Oncology, Hematology, BMT Plus Section Pneumology, University Cancer Center Hamburg, University Medical Center Hamburg-Eppendorf, Hamburg, Germany

**Keywords:** Extragonadal germ cell tumors (EGCTs), Primary mediastinal germ cell tumors, Primary retroperitoneal germ cell tumors, Chemotherapy, Seminoma, Non-seminoma

## Abstract

**Purpose:**

To present the current evidence and the development of studies in recent years on the management of extragonadal germ cell tumors (EGCT).

**Methods:**

A systematic literature search was conducted in Medline and the Cochrane Library. Studies within the search period (January 2010 to February 2021) that addressed the classification, diagnosis, prognosis, treatment, and follow-up of extragonadal tumors were included. Risk of bias was assessed and relevant data were extracted in evidence tables.

**Results:**

The systematic search identified nine studies. Germ cell tumors (GCT) arise predominantly from within the testis, but about 5% of the tumors are primarily located extragonadal. EGCT are localized primarily mediastinal or retroperitoneal in the midline of the body. EGCT patients are classified according to the IGCCCG classification. Consecutively, all mediastinal non-seminomatous EGCT patients belong to the “poor prognosis” group. In contrast mediastinal seminoma and both retroperitoneal seminoma and non-seminoma patients seem to have a similar prognosis as patients with gonadal GCTs and metastasis at theses respective sites. The standard chemotherapy regimen for patients with a EGCT consists of 3–4 cycles (good vs intermediate prognosis) of bleomycin, etoposid, cisplatin (BEP); however, due to their very poor prognosis patients with non-seminomatous mediastinal GCT should receive a dose-intensified or high-dose chemotherapy approach upfront on an individual basis and should thus be referred to expert centers Ifosfamide may be exchanged for bleomycin in cases of additional pulmonary metastasis due to subsequently planned resections. In general patients with non-seminomatous EGCT, residual tumor resection (RTR) should be performed after chemotherapy.

**Conclusion:**

In general, non-seminomatous EGCT have a poorer prognosis compared to testicular GCT, while seminomatous EGGCT seem to have a similar prognosis to patients with metastatic testicular seminoma. The current insights on EGCT are limited, since all data are mainly based on case series and studies with small patient numbers and non-comparative studies. In general, systemic treatment should be performed like in testicular metastatic GCTs but upfront dose intensification of chemotherapy should be considered for mediastinal non-seminoma patients. Thus, EGCT should be referred to interdisciplinary centers with utmost experience in the treatment of germ cell tumors.

## Introduction

Germ cell tumors (GCT) arise predominantly from within the testis, but an important subset of about 5% of the tumors are primarily located extragonadal with no testicular primary tumor being detectable [1].

Extragonadal germ cell tumors (EGCTs) are a heterogeneous group of tumors of neoplastic germ cells arising from extragonadal anatomical locations located in the midline of the body. Primary EGCT are considered a special subgroup of GCT with a poorer prognosis due to larger volume and different biology. They result from malignant transformation of germ cells that were either maldistributed during embryonic development or germ cells that naturally occur to control immunological processes or other organ functions at extragonadal locations [1].

EGCTs include seminomatous tumors (classical seminoma), and non-seminomatous tumors (embryonal carcinoma, teratoma, yolk sac carcinoma, chorioncarcinoma).

Histological, serological and cytogenetic characteristics of EGCTs are similar to those of primary testicular GCT, but differences in clinical behavior suggest that gonadal and extragonadal tumors are biologically different [1].

EGCTs have significantly larger tumor masses at diagnosis. There is a predominance for the occurrence in the anterior mediastinum, especially of non-seminomatous subtypes. A association of EGCTs has been described with Klinefelter syndrome [2]. Furthermore, about 5–10% of patients with non-seminomatous EGCTs of the mediastinum are at risk for the development of acute leukemias, which are not therapy induced but rather biologically associated [1, 3]. These differences may account for the somewhat poorer outcome of some subgroups of patients with EGCTs.

The aim is to present the current evidence on classification, diagnosis, prognosis, therapy and follow-up of EGCT and to highlight recent studies in this special subfield of GCT.

## Methods

This work is based on a former systematic literature search that was conducted for the elaboration of the first German clinical practice guideline [4]. Here, we updated a systematic literature search using the biomedical databases Medline (Ovid) and the Cochrane Library to identify studies on classification, diagnosis, prognosis, treatment and follow-up of EGCT. We considered studies that were published between January 2010 to February 2021 with available full texts publications in English or German language. Study selection, data extraction and risk of bias assessment was done by one reviewer. Relevant and well-known articles published before the search date of 2010 were additionally used to supplement the evidence base, as the cut-off of the year 2010 was formerly chosen due to limited resources in the guideline development process. The Oxford 2009 criteria were used to rate the level of evidence of included studies [5]. Two reviewers assessed the risk of bias in cohort studies with the Scottish Intercollegiate Guidelines Network (SIGN) checklist for cohort studies [6], prognostic studies with the QUIPS tool and for case series [7], we used a self-developed tool based on the quality appraisal checklist of Guo et al. [8]. This systematic review adheres to the recommendations of the PRISMA (Preferred Reporting Items of Systematic Reviews and Meta-analysis) guidelines [9].

## Results

**Fig. 1:**
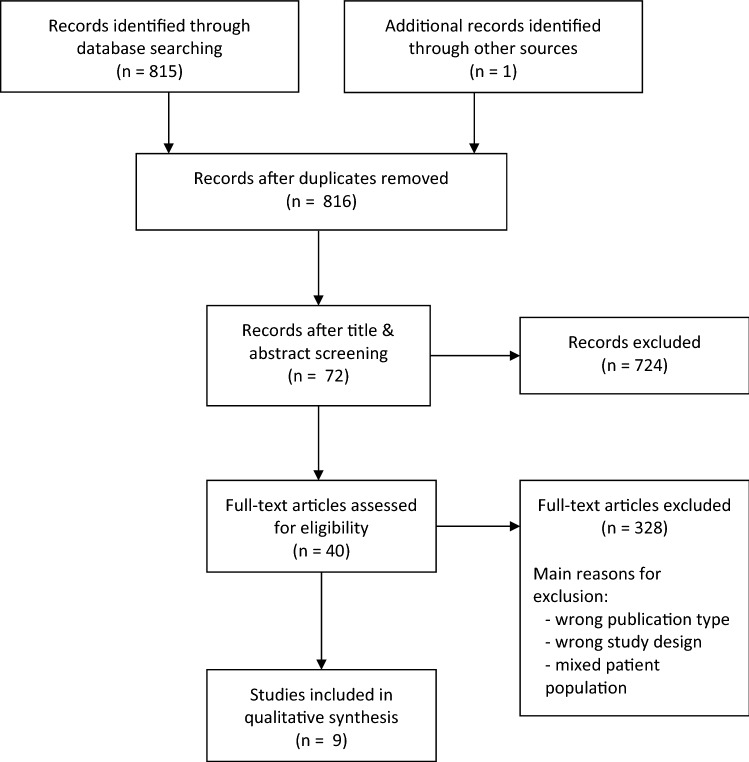
Study flow diagram

The systematic literature search identified nine studies (see Fig. 1). Four of these studies addressed prognostic [10–13] and five therapeutic questions [14–18]. All treatment studies identified were retrospective, whereas four were case series and one was a cohort study (Table 1). Data on ECGT are limited, since all data mainly based on case series, studies with small patient numbers and no comparative prospective studies. Risk of bias of included studies ranged from low to high risk of bias. Missing control of possible confounders, missing information on patient inclusion and insufficient description of interventions were mostly the reasons for assigning a high-risk judgement to a study.

**Table 1:** Table of patient characteristics

References	Design Number of patients Country Follow-up	Objective	Patients	Prognostic factors	Outcome	Statistical analysis	Results	Funding and conflict of interests	Level of evidence Risk of bias *additional comments*
*Prognostic studies*									
Alanee 2014 [10]	Retrospective database study (SEER-database) *n* = 37.283 USA 1973–2008	To examine the effect of extragonadal tumor site on the risk for cardiovascular, hematopoietic malignancies, and solid cancer-related causes of death	*n* = 17.715 nonseminomatous *n* = 19.568 seminomas *n* = 824 (2%) mediastinal GCTs *n* = 1.469 (4%) nonmediastinal extragonadal tumors 94% with gonadal GCTs	effect of primary cancer site	cardiovascular malignancies hematopoietic malignancies solid cancer-related causes of death	Multivariate analysis	Mediastinal Cardiovascular disorders HR 4.49 95% CI 2.52–8.02 *p* < 0.0001 Hematopoietic malignancies HR 8.84 95% CI 3.14–24.73 *p* < 0.0001 Solid cancers HR 1.46 95% CI 0.36–5.90 *p* = 0.59 Nonmediastinal extragonadal Cardiovascular disorders HR 2.75 955 CI 1.67–2.51 *p* < 0.0001 Hematopoietic malignancies HR 0.93 95% CI 0.13–6.84 *p* = 0.94 Solid cancers HR 1.85 95% CI 0.68–5.01 * p* = 0.23	None conflict of interests Supported by the Sidney Kimmel Center for Prostate and Urologic Cancers	LoE 2b low risk of bias
Buchler 2012 [11]	prognostic study *n* = 36 Czech Republic 1994–2010 Median follow-up: 32 mo (5–152 mo)	We have studied the role of fluorodeoxyglucose positron emission tomography for outcome prediction of patients with primary extragonadal germ cell tumors	Primary extragonadal germ cell tumors Median age at diagnosis: 35 y (range 18–66 y)	baseline characteristics: age, presence/ absence of constitutional symptoms, mediastinal versus non-mediastinal primary, seminoma versus nonseminoma, presence/ absence of choriocarcinoma histology, LDH elevation, AFP elevation, HCG elevation, S stage, bulky tumor, lung post-treatment results: FDG-PET response after first-line of therapy FDG-PET response after completion of therapy marker response after first-line of therapy, marker response after the completion of therapy retroperitoneal nodal dissection as a part of treatment	positive FGD-PET as predictor for survival OS	Kaplan Meier curves Cox proportional hazards model	baseline characteristics: none were significantly prognostic for OS post-treatment results: Negative FDG-PET after completion of treatment: 3-ys-OS: 100% 5-ys-OS: 89% Positive FDG-PET after 1^st^ line of treatment: None of the patients survived at three years after diagnosis Positive tumor markers after 1^st^ line of treatment 3-ys-OS: 69% 5-ys-OS: 20% Negative tumor markers after 1^st^ line of treatment 3-ys-OS: 90% 5-ys-OS: 67%	The authors declare they have no potential conflicts of interest Supported by grant G9005 (NS10420-3/2009) from the Department of Health, the Czech Republic	LoE 2b moderate risk of bias possible confounders and statistical values (e.g. confidence intervals) not sufficiently described, not all results of defined outcomes reported
Necchi 2015 [12]	modelling study *n* = 86 1985 -2012 Italy	Intention of building a prognostic model including information on disease and treatment characteristics	Primary mediastinal germ cell tumors Mean age: 29.8 y (15–63 y)	Patient, disease, and outcome characteristics: histologic subtype, type of elevated marker at diagnosis, presence of a mediastinal syndrome (discernible only for cases with face or arm swelling reported on charts) and site of distant metastases, if present	OS	Cox proportional hazards regression analysis	Final multivariate model for OS: Presence of lung metastases HR, 3.03; 95% CI, 1.12–8.15; *p* = 0.028 Combination of surgery with histology viable cancer vs. necrosis and/or Teratoma: HR 6.17; 95% CI 1.31–29.00; * p* = 0.021 necrosis and/or teratoma No versus yes: HR 11.06; 95% CI 2.28–53.56; * p* = 0.003 5y-OS: No Surgery and Presence of Lung Metastases 25.0% (95% CI 7.5–83.0) No Surgery and Absence of Lung Metastases 37.5% (95% CI 19.0–73.8) Surgery, Viable Cancer and Presence of Lung Metastases 25.4% (95% CI 7.7–83.8) Surgery, Viable Cancer and Absence of Lung Metastases 60.6% (95% CI 36.8–99.8) Surgery, necrosis and/or teratoma and Presence of Lung Metastases 75.0% (95% CI 42.6–100.0) Surgery, necrosis and/or teratoma and Absence of Lung Metastases 87.5% (95% CI 67.3–100.0)	The authors have stated that they have no conflicts of interest no information about funding	LoE 2b high risk of bias *only significant results shown*
Rivera 2010 [13]	prognostic study *n* = 31 (2 female) 1986–2009 France	This study tries to determine the prognostic factors of mediastinal germ cell tumors	primary mediastinal germ cell tumors Median age: 28 y (16–60 y)	Age Gender Tumor histological type Extension at diagnosis Tumor markers concentrations Preoperative chemotherapy Markers after first-line chemotherapy Surgical treatment Viable tumor in resected residue	5y-OS	Univariate analysis Multivariate analysis	Multivariate analysis Surgical resection of the tumor OR 5.10; 95% CI 1.49–17.45; *p* = 0.009 5y-OS: surgical treatment: 65.6% no surgical treatment 25% Other variables not statistically significant	No information about conflict of interest and funding	LoE 2b low risk of bias

Reference	Design Number of patients Country Follow-up	Objective	Patients	Intervention	Control	Results	Funding and conflict of interests	Level of evidence Risk of bias *Additional comments*
*Therapeutic studies*								
De Latour 2012 [14]	retrospective case serie *n* = 21 (1 female) 1983–2010 France Median follow-up: 98 mo	We retrospectively assessed surgical outcomes in patients with high serum tumour marker levels after chemotherapy for primary mediastinal non-seminomatous germ cell tumours	primary mediastinal non-seminomatous germ cell tumours Median age: 30 y (19–49 y)	residual tumour excisio* n* + chemotherapy	no control group	Serum tumor makers returned to normal: 11/21 5y-survival rate tumours confined to the mediastinum: 50% extra-mediastinal involvement: 27% *p* = 0.320 with second-line chemotherapy (* n* = 11): 42% without: 30% *p* = 0.61 DFS Median: 5 mo (2–188 mo) 1y-rates: 38% 5y-rates: 33%	None conflict of interest declared No information to funding	LoE 4 at risk of bias No information about consecutive inclusion of the patients and funding
Dechaphunkul 2016 [15]	retrospective case serie *n* = 40 (1 female) 2003–2013 Thailand Median follow-up: 13 mo (1–132 mo)	To review the clinical characteristics and outcomes of patients with mediastinal germ cell tumors treated at our institution between 2003 and 2013	Mediastinal germ cell tumors *n* = 7 seminoma 33 = non-seminoma Median age: 24 y (15–52 y)	Chemotherapy 87% bleomycin, etoposide, cisplatin 13% etoposide and cisplatin	no control group	44% achieved a complete serological response 5y-OS Seminoma: 71.4% Non-Seminoma: 27.3% *p* = 0.051 chemotherapy followed by surgical resection: 72.7% no surgery: 20.7% *p* = 0.02 Loss to follow-up - early discontinuation because of intolerable toxicities (* n* = 3) - disease progression (* n* = 5) - unknown (n = 5)	No author has any financial conflict of interest with respect to this project Supported by the Faculty of Medicine, Prince of Songkla University, Songkhla, Thailand	LoE 4 low risk of bias
Liu 2011 [16]	retrospective cohort study *n* = 55 (3 female) 1988–2010 China Median follow-up: 31.4 mo	The aim of this study was to evaluate the clinical characteristics and survival outcomes of patients with primary mediastinal germ cell tumor by identifying the prognostic factors and efficacies of different treatment modalities	primary mediastinal germ cell tumor *n* = 38 seminoma *n* = 27 non-seminoma Mean age: 24.65 y (12–64 y)	Different analysis Treatment scenarios three treatments: surgery, chemotherapy, radiotherapy two treatments: chemotherapy + surgery or radiotherapy one treatment: surgery or chemotherapy •Resection •Radiotherapy		OS Treatment scenarios Three treatments (* n* = 11): 83.26 mo Two treatments (* n* = 25): 118.27 mo One treatment (* n* = 17): 48.63 mo No Treatments (* n* = 2): 4.65 mo *p* = 0.000 Resection Complete (* n* = 22): 96.23 Incomplete (* n* = 7): 55.36 mo No resection (* n* = 26): 48.20 mo *p* = 0.031 Radiotherapy with (* n* = 23): 111.04 mo without (* n* = 32): 66.56 mo *p* = 0.026 Prognostic factors of poor OS (multivariate analysis) - extensive extent (* p* = 0.012) - poor response rate at initial evaluation (* p* = 0.002)	None conflict of interest No information about funding	LoE 4 RoB: not acceptable No comparison of patient characteristics between intervention, treatments are not clearly described; No confidence intervals given
Rodney 2012 [18]	retrospectie case serie *n* = 34 1998–2005 USA	We retrospectively assessed treatment outcomes at a single institution	primary mediastinal germ-cell tumors *n* = 7 seminoma *n* = 27 non-seminoma Mean age: Seminoma: 32 y (20–60 y) Non-Seminoma: 30 y (20–53 y)	Chemotherapy		Non-seminoma patients - 13 were alive at median OS 33.5 mo - 7 were recurrence-free at a median 56.5 mo Seminoma patients were alive and free of disease Patients with progressed disease (* n* = 5) and need salvage treatment (* n* = 14) 3y-OS: 23% PFS was associated with absence of risk factors	No information about conflict of interest supported by the National Cancer Institute at the National Institutes of Health	LoE 4 at risk of bias dosage of the chemotherapy regimens has not been described, no information about consecutive inclusion of the patients and conflict of interest, descriptive analysis
Sarkaria 2011 [17]	retrospective case series *n* = 57 1980–2008 USA Median follow-up 5.3 y	The purpose of this study was to analyze a single institution’s surgical experience with Primary Mediastinal Non-Seminomatous Germ Cell Tumors	Primary Mediastinal Non-Seminomatous Germ Cell Tumors Median age 30 y (18–50 y)	Surgery and platinum-based preoperative chemotherapy (54/57)		OS Median: 31.5 mo 2y: 56% PFS Median: 9.1 mo 2y: 46% Predictors for worse OS (multivariate analysis) increasing preoperative tumor markers (HR 3.2; 95% CI 1.1–9.4) * p* = 0.04	The authors declare no conflicts of interest No information about funding	LoE 4 at risk of bias dosage of the chemotherapy regimens has not been described, no information about consecutive inclusion of the patients and funding

*DFS* disease-free survival, *GCT* germ cell tumor, *HR* hazard ratio, *LoE* level of evidence, *OS* overall survival, *PFS* progression-free survival, *SEER-database* Surveillance, Epidemiology and End Results database, *RoB* Risk of Bias, *RFS* recurrence-free survival, *USA* United States of America, *y* years

### Localizations of EGCTs

EGCTs are localized primarily mediastinal or retroperitoneal, but also at any other site along the midline except the testis [19]. The anterior mediastinum (50–70%) and retroperitoneum (30–40%) represent the most common locations of EGCT [20]. Less common sites of origin for EGCTs are the pineal gland (glandula pienalis), os sacrum, prostate, orbita, urinary bladder, or liver (Table 2). Epidemiological data from Germany show a high preference for the brain, pituitary and pineal gland with about 40% of patients (61 of 157 patients) [21]. According to a study by the National Cancer Register of Finland, the incidence of EGCTs is about 0.18/100.000 [22].

**Table 2:** Locations of extragonadal germ cell tumors (EGCTs) [1]

Common Mediastinum Retroperitoneum Pineal and suprasellar regions Sacrococcyx (infants and young children only)
Very rare Prostate Liver and gastrointestinal tract Orbita

#### Mediastinal EGCTs

In a case series, 320 male patients with confirmed primary mediastinal EGCTs were reported [23]. The histological discrimination between pure seminoma, non-seminoma and teratoma is very important. Teratomas and pure seminomas are the most common histological subtypes of mediastinal EGCTs. Mature mediastinal teratomas are considered “benign” and are treated by surgical resection alone, as chemotherapy or radiotherapy are not effective. About 43% of all mediastinal tumors harbor parts of a teratoma [24]. About 63% of them are mature teratomas, 37% are teratomas with malignant transformations, for example GCT plus sarcoma components or adenocarcinoma components [24]. Teratomas with malignant GCT components, for example seminoma, embryonal carcinoma or yolk sac tumor are considered as malignant non-seminomatous EGCTs. Unlike conventional GCTs, which usually respond favorable to platinum-based chemotherapy, teratoma with malignant transformation is a very aggressive tumor that is resistant to chemotherapy and needs extended surgical treatment [4, 25].

Mediastinal EGCTs are differentiated into seminomas and non-seminomas. Among mediastinal EGCTs, seminomas account for 40% of the non-teratoma EGCT and are thereby more common than in EGCTs in general, where they account for only 20–24% of the tumors. In a large international case series of 635 patients with mediastinal and retroperitoneal EGCTs, 104 patients showed pure seminomas and 524 had non-seminomatous tumors [26]. In contrast to testicular non-seminomatous GCTs, mediastinal non-seminomatous EGCTs contain embryonal carcinoma less frequent and yolk sac tumor components more frequent. In a series of 64 patients, histology revealed a pure yolk sac tumor in 60% of the patients, a chorionic carcinoma in 12%, and a pure embryonic carcinoma in about 9% of the patients [27].

#### Retroperitoneal EGCTs

Retroperitoneal EGCTs have a clinical behavior very similar to that of testicular GCTs [24]. The genesis of retroperitoneal GCTs is still under debate [20]. Undisputed is an association between a premalignant testicular lesion and retroperitoneal EGCT. The differential diagnosis to “burnt out” EGCT of the testis with retroperitoneal metastasis is difficult. A retrospective analysis from Switzerland of 26 patients with a retroperitoneal EGCT discovered pathological findings on clinical examination of the testes in 11 patients (42%) [28]. 14 patients (54%) showed a testicular atrophy and/or induration, one patient had an enlarged testicle. Ultrasound examination demonstrated a suspicious lesion in every patient. Finally, pathological review of the testicular tissue was performed for 25 of the 26 patients. They revealed scar tissue in 12 patients (48%), intratubular neoplasia in 4 (16%) and vital malignant tumor in 3 patients (12%). Conclusively, the authors postulated that primary EGCT in the retroperitoneum are very likely a rare or non-existing entity and should be considered as metastases of a viable or burned-out testicular cancer until proven otherwise [28].

#### Other rare localizations of EGCTs

Less frequent sites of EGCT are the pineal gland (glandula pienalis), os sacrum, prostate, orbita, urinary bladder or liver [19]. Epidemiological data from Germany indicate a high preference for the brain, the pituitary and pineal gland accounting for 40% of the patients (61 of 157 patients) [21]. In adults, mature teratomas are the most common presentation of sacrococcygeal EGCTs, but GCTs without teratomatous components have also been documented [29].

Pathology of EGCTs**

Primary EGCTs are considered a special subgroup of GCTs. They result from malignant transformation of germ cells that were either maldistributed during embryonic development or from germ cells that naturally occur at extragonadal sites for the purpose of controlling immunological processes or other organ functions [30, 31].

In principle, the same histological subtypes are present in EGCT as in testicular localized GCTs (seminomas and non-seminomatous EGCT). EGCTs include seminomatous tumors (classical seminoma), and non-seminomatous tumors, including embryonal carcinoma, teratoma (mature or immature), yolk sac carcinoma and chorioncarcinoma. EGCTs constituted by two or more histotypes are referred to as mixed germ cell tumors, which classified as non-seminomatous tumors. In mediastinal EGCT, seminomatous tumor components and teratoma components are frequently detectable [26, 27].

There is a clear association between EGCT and Klinefelter’s syndrome, a male genetic disorder characterized by the 47, XXY karyotype, small and soft testis, sterility, eunuchoid habitus, gynecomasty, high levels of FSH and a 20-fold increased risk for breast cancer. Regarding the increased risk of EGCT in Klinefelter´s patients, it is still unclear whether the development of GCTs in patients with Klinefelter’s syndrome is the result of a primary genetic abnormality or of an abnormal hormonal milieu that primes premalignant tumor cells into malignant tumor development [1].

In addition, an increased rate of yolk sac components, elevated AFP levels, and TP53 aberrations are observed in EGCTs compared to testicular GCTs [32].

Primary mediastinal non-seminomatous EGCTs can be the origin of hematologic neoplasms with an incidence of 5–10% [33]. These hematological neoplasms frequently contain an isochromosome 12p, which is the cytogenetic hallmark of GCTs and confirms the common biological background of both the EGGCT and the hematological neoplasia [34]. Hematologic malignancies associated with primary mediastinal non-seminomatous germ cell tumors are mainly disorders of the megakaryocyte lineage characterized as acute mega-karyoblastic leukemia (AML-M7) and myelo-dysplastic syndrome with abnormal megakaryocytes [33].

### Clinical symptoms and diagnosis

EGCTs often present only at advanced stages due to tumor-related symptoms, but they also occur as incidental findings during diagnostic or other therapeutic interventions. The clinical presentation of EGCT varies widely. Avery advanced cases of mediastinal EGCT may present with pulmonary symptoms or venous compression syndromes (including superior vena cava syndrome). When the EGCT is primarily located in the retroperitoneum, abdominal pain, back pain, weight loss, inferior vena cava thrombosis, or hydronephrosis are the main clinical presentations.

The most conclusive data regarding clinical symptoms were shown in 2012 in the largest published series of EGCTs with 635 patients by Bokemeyer et al. [26]. Patients with mediastinal EGCT had in particular dyspnea (25%), chest pain (23%) and cough (17%) at initial presentation, followed by fever (13%), weight loss (11%), vena cava occlusion syndrome and fatigue/weakness (6%). Less frequent symptoms than expected were enlarged cervical lymph nodes (2%), hemoptysis, hoarseness and dysphagea (1% each).

In patients with a primary retroperitoneal EGCT, the main symptoms were abdominal (29%) and back pain (14%), followed by weight loss (9%), fever (8%), vena caval or other thrombosis (9%), palpable abdominal tumor (6%), enlarged cervical lymph nodes (4%), scrotal edema (5%), gynecomastia and dysphagea (3%).

The symptoms of EGCT patients are caused by the growing tumor mass. After appropriate imaging with ultrasound and computed tomography/magnetic resonance imaging (CT/MRI), the diagnosis should be confirmed histologically. Depending on the localization, this can be performed by fine-needle aspiration cytology, percutaneous biopsy or specimen resection during mediastinoscopy/laparoscopy [1]. In addition, the evaluation of serum tumor markers (AFP, beta-hCG, LDH) is required for the correct diagnosis and classification of EGCTs according to the International Germ Cell Cancer Collaborative Group (IGCCCG) [35].

The role of FDG-PET-CT in the primary diagnosis of EGCT is unclear. However, in evaluating the success of first-line systemic therapy, FDG-PET may have a decisive value. Additionally, Buchler et al. showed that a negative FDG-PET after the completion of EGCT treatment was a powerful predictor of long-term survival with 100% of the patients surviving three years and 89% surviving five years after diagnosis [11].

Retroperitoneal EGCTs are often associated with a "burned-out" tumor of the testis, whereas in mediastinal EGCTs, this is extremely rare [36]. The question whether a clinical and sonographic non-suspicious testis has to undergo histological assessment is still controversial. In the largest international EGCT series of Bokemeyer et al., about 11% of the patients underwent a testicular biopsy. In 3% of the cases, a Sertoli cell-only syndrome was diagnosed, 31% had atrophic or fibrotic testicular tissue and only 9% germ cell neoplasia in situ (GCNIS) lesions. Current guidelines do not recommend the removal of the testis as long as the ultrasound findings are normal [4].

In 2001, Hartmann et al. observed that metachronous testicular GCTs most commonly occurred in seminomatous EGCTs with a cumulative risk of 10% within 10 years [34]. This risk appeared to be higher than in other series of metastatic GCTs. Thus, a routine testicular biopsy in EGCT patients was discussed. However, these secondary testicular tumors are quite easy to detect and, especially in the case of seminoma, highly curable. Therefore, a routine bilateral testicular biopsy in EGCT patients cannot be routinely justified. However, regular ultrasound of the testis during follow-up seems reasonable. Due to the limited number of cases, clear evidence-based recommendations cannot be given regarding the discussed issues.

### Classification

In the largest international analysis of EGCTs, patients with seminomatous EGCTs with the primary localization in the mediastinum and retroperitoneum had an equivalent prognosis to patients with primary testicular seminoma according to the IGCCCG classification (Table 3) and similar metastatic locations [26, 37]. In spring 2021, the IGCCCG update consortium improved the 1995 classification by developing and independently validating a more detailed prediction model. This model identified a new cut-off of lactate dehydrogenase at a 2.5 upper limit of normal and increasing age and presence of lung metastases as additional adverse prognostic factors. Overall, the long-term outcome of patients from all prognostic categories was improved compared to 1995, however, mediastinal non-seminoma remained a clear criterion for “poor prognosis”. An online calculator is provided (https://www.eortc.org/IGCCCG-Update) [37].

**Table 3:** Prognostic-based staging system for metastatic germ cell cancer—International Germ Cell Cancer Collaborative Group (IGCCCG)—Update 2021 [35, 37] Online calculator: https://www.eortc.org/IGCCCG-Update

Good-prognosis group 5-year PFS 90% 5-year survival 96%	
Non-seminoma	*All of the following criteria:* Testis/retroperitoneal primary No non-pulmonary visceral metastases Age AFP < 1000 ng/mL hCG < 5000 IU/L (1000 ng/mL) LDH < 2.5 × ULN
Seminoma	*All of the following criteria:* Any primary site No non-pulmonary visceral metastases Age Normal AFP Any hCG Any LDH
Intermediate-prognosis group 5-year PFS 78% 5-year survival 89%	
Non-seminoma	*Any of the following criteria:* Testis/retroperitoneal primary No non-pulmonary visceral metastases Age AFP 1000–10,000 ng/mL or hCG 5000–50,000 IU/L or LDH 2.5–10 × ULN
Seminoma	*All of the following criteria:* Any primary site Non-pulmonary visceral metastases Age Normal AFP Any hCG Any LDH
Poor-prognosis group 5-year PFS 54% 5-year survival 67%	
Non-seminoma	*Any of the following criteria:* Mediastinal primary Non-pulmonary visceral metastases Age AFP > 10,000 ng/mL or hCG > 50,000 IU/L (10,000 ng/mL) or LDH > 10 × ULN
Seminoma	No patients classified as “poor-prognosis”

The same parameters which identified patients with testicular seminomatous tumors, also predicted the individual prognosis in seminomatous tumors of primary extragonadal origin. Therefore, patients with seminomatous EGCTs should be classified as either "good prognosis" or "intermediate prognosis" according to IGCCCG and treated accordingly.

In the case series reported, non-seminomatous EGCTs had a worse prognosis than seminomatous EGCTs with a 5-year survival rate of 62% for retroperitoneal and 45% for mediastinal non-seminomatous EGCTs [26]. The analysis clearly indicated that mediastinal EGCTs belong to the poor prognosis group even if they otherwise fulfilled the IGCCCG criteria of good or intermediate prognosis. All patients with mediastinal non-seminomatous EGCT are classified as poor prognosis irrespective of further metastatic spread or serum tumor marker levels [38].

Patients with retroperitoneal non-seminomatous EGCTs are classified according to the serum tumor marker constellation of the IGCCCG classification and are treated analogously to metastatic testicular non-seminomatous GCTs. Several other studies corroborated these results [39–42].

### Treatment

#### Seminomatous EGCTs

Patients with seminomatous EGCTs should be treated according to the IGCCCG classification prognostic group, with three cycles of bleomycin, etoposide, cisplatin (BEP) for good prognosis and four cycles of BEP for intermediate prognosis patients. An alternative chemotherapy regimen in case of contraindications to bleomycin in good prognosis patients is four cycles of etoposide, cisplatin or substituting bleomycin by ifosfoamide if a subsequent pulmonary operation is planned [41, 4]

EGCT patients with pure seminomatous germ cell tumors have a better prognosis than non-seminomas, especially because seminoma cells are highly susceptible to cisplatin-based chemotherapy and ionizing radiation. A residual tumor resection is not routinely required. In three retrospective studies of patients with mediastinal seminomatous EGCTs (case numbers ranging from 7 to 17), 5-year overall survival (OS) rates of 71% and 100% were reported [15, 16, 18].

In a case series of 52 patients with a retroperitoneal pure seminoma and 51 patients with a mediastinal pure seminoma, the 5-year progression-free survival (PFS) and the 5-year OS rates were 87% and 90%, respectively [43]. 75% of the patients were successfully treated with chemotherapy alone.

Similar to the therapy of testicular CS II GCTs, retroperitoneal seminomatous EGCTs can be treated with radiotherapy if tumor extension is limited. Unfavourable prognostic factors for pure seminomas are the presence of liver metastases or metastases in two or more different organs; however, this leads to a classification of intermediate prognosis in these cases, as seminomas, both metastatic from testicular origin or extragonadal are never categorized as IGCCCG “poor prognosis” [43].

#### Non-seminomatous EGCTs

The standard chemotherapy regimen for patients with a mediastinal non-seminomatous EGCT consists of four cycles of cisplatin-based combination chemotherapy either BEP or PEI. Only few studies with limited numbers of cases have been reported on primary mediastinal non-seminomatous EGCTs [12–14, 18, 42]. In the study by De Latour et al., all 21 patients were treated with first-line chemotherapy, and 52% of patients required second-line chemotherapy. The 5-year OS of patients with tumors confined to the mediastinum was 50% and of patients with extra-mediastinal involvement 27% [14]. A Memorial Sloan Kettering Cancer Center series reported on 57 resected patients with primary mediastinal non-seminomatous GCT, 54 of whom were pretreated with platinum-based chemotherapy [17]. Median OS was 31.5 months, and preoperatively normalized or reduced serum tumor markers after chemotherapy were the strongest predictors of improved survival [17].

In non-seminomatous EGCT, residual tumor resection (RTR) should be performed analogously to metastatic testicular GCT after the completion of chemotherapy. In primary mediastinal non-seminomatous EGCT resection of all visible residuals (even < 1 cm) should be aimed for, and post-chemotherapy elevated serum tumor markers should not discourage surgery. In this context, some studies performed multivariate analyses, all of which emphasized surgical therapy after initial systemic therapy as an important prognostic factor [12, 13].

No prospective studies have investigated the role of high-dose chemotherapy in the first-line treatment of extragonadal germ cell tumors so far. In a meta-analysis of 524 patients with non-seminomatous germ cell tumors in 2002 by Bokemeyer et al., 59 patients (13%) were treated with high-dose chemotherapy [26]. In the univariate analysis, high-dose chemotherapy was not a significant prognostic factor for improved survival. In contrast, in another study [44] with 64 EGCT patients out of 235 patients with mainly poor prognosis criteria (IGCCCG) treated with initial high-dose chemotherapy, 5-year overall survival was reported to be higher compared to standard-dose platinum-based combination chemotherapy with four cycles of BEP (82% versus 71%) [44]. Due to very poor prognosis of patients with primary mediastinal EGCT, a dose-intensified or high-dose chemotherapy should be chosen primarily and is recommended in the German national S3 guideline for testicular cancer.

In contrast to all other non-seminomatous EGCTs, mature teratomas are resistant to chemotherapy. If there is clear histologic evidence of a mature teratoma and no elevation of serum tumor markers for GCTs, surgical resection of the EGCT is the best therapeutic option.

In summary, all patients with mediastinal non-seminomatous EGCTs are classified in the poor prognosis group. Furthermore, the chemosensitivity of primary mediastinal non-seminomatous EGCTs appears to be lower compared to testicular and/or retroperitoneal EGCT, as vital carcinoma portions are frequently found in resected post-chemotherapeutic ETGC mediastinal residuals [14, 45]. Accordingly, upfront intensification of therapy with high-dose chemotherapy and surgical resection of all visible residuals after chemotherapy should be preferred. The chance of cure even when employing high-dose therapy plus autologous stem cell support as salvage therapy is very limited with long-term survival of only 11% in relapsed mediastinal EGCT patients [46]. The complex management of these patients should be performed in experienced centers.

#### Prognosis and follow-up

Non-seminomatous mediastinal EGCT have a poor prognosis with a 5-year OS of 40–45%, with inferior response rates to chemotherapy, especially in recurrence. The prognosis of retroperitoneal EGCT is better and similar to that of metastatic testicular GCT. The literature search for the follow-up of EGCT did not yield any relevant results. It is important to note that patients presenting with poor prognosis EGCT should be followed-up individually by specialized centers. The follow-up intervals of clinical examinations, ultrasound of the testicles, determination of tumor markers and radiological examinations (MRT/CT scans) should be similar to those of patients with metastatic GCT but must be adapted to the individual needs of the patient. EGCT patients have an increased risk for death from cardiovascular disease and those with mediastinal non-seminomas for the development of hematopoetic malignancies compared to testicular GCTs. These aspects need to be considered during follow-up. However, as many patients with EGCT can be long-term cured, they should be included into specific testicular cancer survivorship programs [10].

## Discussion and conclusion

EGCTs are a very rare tumor entity with specific biological and clinical features. In the case of seminomatous histology, the prognosis is best represented by the IGCCCG classification, regardless of the location of the EGCTs, either in the mediastinum or retroperitoneum. However, the majority of EGCT patients have non-seminomatous components. Patients with retroperitoneal non-seminomatous EGCT should also be treated according to the IGCCCG risk classification. However, patients with mediastinal non-seminomatous EGCT are always classified as “very” poor prognosis. Upfront high-dose chemotherapy appears to be the best therapeutic option for these patients, since the chance of survival using an effective salvage chemotherapy in case of relapse is extremely low. Performing residual tumor resection (RTR) should be the standard procedure for non-seminomatous EGCT patients after initial chemotherapy, especially in patients suffering from mediastinal EGCTs, whenever technically feasible. Because of their poor prognosis and several potential clinical problems associated with the disease and treatment, these patients should be treated and followed-up in specialized centers.

Due to the rarity of EGCT cases, a detailed analysis in prospective randomized trials is hardly possible, so that the assessment of the best diagnostic and therapeutic options has to be based on retrospective studies.

The problem with the current systematic review is the lack of validated data from the last 10 years. Few statistically relevant studies in recent years could be identified, so important studies from earlier decades were also included in the review process.

## Data Availability

Yes.
